# A Treatise on the Nature, Symptoms, Causes, and Treatment of Insanity, &c. &c.

**Published:** 1838-07-01

**Authors:** 


A TREATISE ON THE NATURE, SYMPTOMS, CAUSES AND TREATMENT OF INSANITY, &c. &c. By Sir *W. C. Ellis*, M.D. Resident Medical Superintendant of the Asylum at Hanwell, &c. 8vo. pp. 342.

IF large practical experience could unravel the mysteries of the brain and nervous system, and disclose the connexion between mind and matter in this sublunary state of our existence, the author of the work under review would have strong claims on our attention. Previously to his residence at Hanwell, he belonged to the asylum at Wakefield, where the sphere of his observation must have been extensive—while the ample field of experience in the Middlesex establishment must have afforded him all the opportunities which an explorer of the melancholy malady under consideration could wish. But what can be added now to the etiology, pathology, or treatment of insanity, after the numerous works which have been published on the subject? That there is yet much to learn in the investigation of insanity, our author himself acknowledges with regret; but the question is two-fold—are we likely to pry much deeper into the insane mind—and if we are, has the present work enabled us to do so? The author very modestly states as follows:

"Though my attention, from early life, has been particularly directed to Insanity, and a residence in the Asylums at Wakefield and Hanwell, during nearly twenty years, has placed under my immediate care and observation upwards of 2,700 cases, I feel that I have still much to learn. Even if the general view taken in the present work be correct (as I fully believe it to be), patient subsequent investigation will be required to make the picture in all its parts complete. Should I succeed in exciting an interest on the subject at all adequate to its importance, it will soon be investigated by men of more leisure, deeper research, and greater anatomical skill, than myself. If the end be but answered, and the Insane benefited, I care not whether it be by the adoption of the plan mentioned in the following pages, or by any other means." vii.

But from a well-experienced writer like the present, there will always be much gleaned, of a practical nature, that will repay the perusal of all those interested in the management, at least, of insanity. We shall glance very rapidly, however, over the greater portion of the work, because it consists chiefly of a great number of valuable cases and illustrations, which cannot be analyzed.

The first Chapter treats of the nature of insanity, and we are glad that Dr. Ellis (we drop the knighthood for brevity's sake) takes the more rational doctrine that insanity is a corporeal disease or disorder *i. e.* of the brain.

"In carefully looking over the *post mortem* reports of those whose cerebral organization I have examined, I find that in 154 male patients, 145 had disease very strongly marked, either in the brain or the membranes. Of the nine remaining, two were idiots from birth; one died of dysentery, another of epilepsy; the other five cases had not been insane more than a few months, and died of other diseases. Of the females, sixty-seven were examined; and sixty-two found with disease in the brain or membranes: in the other five, no disease was to be discovered. Two of these were idiots from birth, and, with one exception, the others recent cases." 20.

The particulars of several of these cases are given. In support of the opinion that increased vascular action is present at the commencement of insanity, Dr. Ellis offers the following case.

"The deceased was thirty-five years of age, and he had only been insane a few months at the time of his death. On dividing the scalp, a considerable quantity of blood escaped; on removing the dura mater, the whole surface of the brain appeared inflamed, the minutest vessels being highly injected with red blood; the tunica arachnoidea was slightly opaque, in small patches; the substance of the brain was firm; not more than the natural quantity of fluid was found in the ventricles. It will be observed, that in some of those cases no traces of disease in the brain could be discovered. We cannot, however, conclude from this that no disease in the brain existed. We know that diseased action may continue in various parts of the body for a considerable period, and yet not be discoverable by any anatomical investigation. The most skilful anatomist cannot find out by dissection any traces of tic doloureux, cramp, rheumatism, &c. In like manner, a man may have had, for many successive years, attacks of gout, and may ultimately die whilst suffering acutely from the disease, and yet no trace of it having ever existed may be discoverable on the minutest dissection, although, in most instances, it produces, after a time, chalky concretions and distortions of the limbs." 23.

But our author wisely abjures the tenet that the brain is always—or perhaps generally, the primary seat of the *cause* of insanity. The disorder may be in the stomach originally, and the brain sympathising with the stomach, leads to mental derangement. Dr. Ellis need hardly have taken the trouble to combat the absurd notion that "insanity is purely a disease of the mind itself" adverted to by some visionaries—and also by the late Dr. Halloran, though a practical man. Dr. E. next adverts to the *extent* of mental aberration which is necessary to constitute insanity, and to render the individual subject to control. The following opinion is, we think, perfectly correct.

"An individual may erroneously think that he sees various forms and substances, which do not exist except in his own imagination; but as long as his reason is sufficient to correct these false impressions, and he is himself conscious that they have no real existence, he is not a fit subject for confinement. Nay more; even if his reason be not sufficient to correct these false impressions, if they be of such a nature as not to interrupt his ordinary pursuits, or to render him obnoxious to society; as, for instance, if he imagines that he sees and converses with spirits, but is not influenced by them, it would be unjust to lock him up in a madhouse: though it is almost unnecessary to say, that it is of the highest importance that, in both instances, proper steps should be immediately resorted to, before these erroneous impressions have been too much confirmed by time to be incapable of removal. For although in the first instance these effects may be harmless, yet, viewing them but as the symptoms and result of diseased action of the brain and nervous system, which may, if allowed to continue, cause organic disease; it is evidently desirable to use the most expeditious means to restore a healthy state of action in these organs. But if the diseased perceptions be of such a kind as to render him incapable of the management of his affairs, or to make his conduct injurious either to himself or to others, confinement ought immediately to be resorted to." 34.

In the third, or etiological chapter, our author touches on the subject of hereditary disposition to insanity. Out of 1380 patients, there have been 214 whose parents or relatives Dr. E. ascertained to have been previously insane. In 125 of these cases, no other cause could be assigned for the disease. In 65, there were strong moral causes, in conjunction with this hereditary disposition—and in 24 there had been blows on the head preceding the attack. Our author indeed considers this last as amongst the most frequent of physical causes. Old age itself becomes a primary cause of insanity—the brain, like some other organs of the body, being the first to give way in function. Loss of memory, defective judgment, diminished powers of reasoning, altered views, &c. are the common precursors of acute insanity.

"By far the most general primary cause of diseased action of the brain, and therefore of insanity, is over-exertion. When the brain has been for too long a time intensely employed upon any subject, it is thrown into such a state of excitement that its operations are no longer under the control of the will: the incipient stage of insanity then commences, a superabundant flow of blood is propelled to the head, irritation and want of sleep are the immediate consequences, and, if proper treatment be not applied, inflammation is the ultimate result. This diseased action, if unchecked, produces diseased organization, or that chronic state of insanity which is attended by congestion of the vessels, the opacity of the membranes, and serous effusion under them and in the ventricles, so generally found in the heads of those who have been insane for any length of time." 58.

Anxiety of mind, including all the various grades of perturbation, from ecstatic joy to grief profound, is, perhaps, the greatest of all moral causes. Masturbation partakes of a moral and physical nature—and leads, in many cases, to insanity.

The fourth chapter, on the symptoms of insanity, must be only cursorily noticed. One of the first symptoms is a confusion of the intellectual faculties—the senses appearing benumbed—with embarrassment of speech, &c. As the organic disease advances, we observe torpor of the limbs—indisposition to muscular exertion—with congestion and coldness of the lower extremities—gradual emaciation, till at last, death terminates the automatic existence! The following passage deserves attention.

"Intense abstraction of mind may be considered as the first alteration that is observable in the great majority of patients who become insane from moral causes. The ordinary duties of life are either altogether neglected, or only performed upon the pressing solicitation of friends. After this state has continued for a short time, it becomes necessary, if we wish to arrest the attention of the patient, to speak to him loudly and repeatedly; and when at last he seems conscious of what is said, he appears as if just aroused from a dream, and relapses into the same state of forgetfulness, as soon as the sound of the voice has ceased to vibrate in his ears; his whole air and manner evidently indicate that the inner man is dwelling upon a subject far different from that about which he is being addressed. The general desire to please no longer influences the character, and the dejected looks, and the forlorn dress, sufficiently proclaim that the mind is entirely absorbed in its own contemplations.

This is the period when the alarm of friends ought to excite them to the most active measures; this is the time when the advice of a physician is truly desirable. There is now an opportunity of resorting with success to measures, which will prevent the coming on of a malady, the treatment of which is at all times difficult, and which, if neglected at the commencement, is attended with circumstances the most painful to the patients and to their friends, and too frequently sinks the unhappy sufferers into a state of hopeless wretchedness, from which no remedies whatever seem able to release them." 107.

When the brain has been affected by derangement of the digestive organs, one of the most common symptoms is a constant suspicion—the individual always fancying that people are combining against his happiness—especially some of his most intimate friends. All attempts to reason him out of his monomania are futile. Religious monomania almost always takes the form of conviction of eternal punishment. It is needless to say that such conviction is almost invariably found amongst those who were most moral and religious previously. Among the lower orders of society—such as come into public asylums—our author has found the fear of witchcraft, a monomania by no means uncommon. The patients are very seldom cured. Another curious and frequent form of insanity is a belief that a venereal taint lurks in the constitution. To a patient of this description, bread-pills were administered, and they produced a copious salivation! They were discontinued, and the salivation ceased. Listen to this ye credulous advocates and disciples of Hahnneman and Dupotet. Surely if imagination can excite so purely physiological an operation as profuse ptyalism, the same agent may induce the hysterical tom-fooleries of Wigmore-street and ——— ———, where they ought never to have been enacted!

Monomaniacs disposed to suicide have their periods of convalescence and exacerbation—sometimes complete temporary immunity from the propensity. This impulse, in our author's experience, is seldom the result of momentary, but of premeditated determination. The mode of commission having been once determined on, the monomaniac will seldom terminate his existence by any other mode. Thus a man having made up his mind to hanging, may be, for weeks or months, within reach of razors, knives, poison, wells, or ponds; but will not commit suicide by means of any of these; till he finds an opportunity for suspension. This is a fact of considerable practical importance. It has often been remarked that phthisis and insanity alternate with each other. The tendency to suicide not infrequently comes on in the last stage of consumption—and many terminate life by their own hands, when a few days or weeks would have brought them to the final goal! There is a peculiar and sinister cast of countenance to be observed in most suicidal maniacs—and also a peculiar fætor about their persons. General despondency and great abstraction, however, are the most common phenomena to be remarked amongst those who are meditating self-destruction. The following passage is curious, and perhaps contains much truth.

"In a state of sanity the various feelings and propensities are kept under control, partly by their mutual influence upon each other, partly from moral causes, and partly from the restraints imposed by society. And where careful education and religious feeling have rendered their due regulation habitual, strong propensities may exist unknown and unsuspected, except by the individual. Now insanity does not create any new class of feelings or propensities. It is, I am aware, a very common opinion, that persons, in consequence of their becoming insane, acquire a new set of faculties, and especially that they become endowed with a great share of cunning. This is quite an error. There is no doubt but that this faculty may be often found very powerfully and actively developed amongst them; but where this is the case, it must have existed in the character previously to the disease coming on. A great number of the patients in public asylums, so far from being particularly cunning, possess no fraudulent dexterity of any kind. The mode in which insanity acts, is to cause an alteration in the mental manifestations and in the conduct, by exciting some to undue exercise, and not permitting others to have their proper influence. Where the passions are thus over-excited, and the controlling feelings are not in sufficient activity, we have necessarily the results previously mentioned; nor ought they to excite in us any surprise, even when observed in the most virtuous and amiable." 127.

Dr. Ellis tries to account for the antipathies which the insane often take towards their best friends and nearest relations, but not, we apprehend, with complete success. We do not think this estrangement is always dependent on, or coupled with an idea that these friends or relations have been accessary to the restraints imposed on the unhappy patient.

"The bodily symptoms, which occur so frequently in insanity as really to deserve to be considered as characteristics of the disease, are very few. The unhealthy action in the brain and its membranes is visible, rather from the alteration in the mental manifestations, than from any uniform corporeal change. In the early stages it is usually marked by irregularity of the secretions, yet it often happens, even in this stage, that, after it has continued for a short time, no alteration whatever takes place in the pulse, and all the secretions appear to be healthy. This is particularly the case where the symptoms denote only a small portion of the brain to be diseased, and where this disease has come on very gradually, the nervous system seeming to accommodate itself to the change, without being so irritated as to disturb the functions of the other parts of the body. And when the derangement has become chronic, it is a well-known fact, that many of the patients, for years together, enjoy excellent bodily health, and exhibit no marks of disease except mental delusions. It is probably this circumstance, which has led to the erroneous notion that medicine is of no use in all cases of insanity. It is singular that this uniformly good bodily health is rarely found, except in those cases where the hallucinations of the patient are confined to one subject." 129.

Where the mental derangement is general, the bodily symptoms are more numerous, as debility, &c. In most instances there is preternatural heat about the head, quickened pulse, &c. The heat of head is generally accompanied by cold feet—want of sleep—clammy perspiration—peculiar fætor. When this last symptom exists, Dr. E. thinks it invariably denotes organic disease of the brain. We can assure our author that we have distinctly noticed this peculiar fætor in people who have completely recovered from insanity, and who have now been in good health for years, We do not dispute the fact, however, of its being a bad symptom. In all those who died with this phenomenon there was found water in the ventricles of the brain. The fætor may be much relieved by the frequent employment of the warm bath. A great want of nervous sensibility is another striking feature of insanity. Great diseases will progress without their being ever complained of by the patient. The very opposite state of the nervous system, however, sometimes obtains. Great hunger is a striking feature—and strange to say, the very reverse, or total anorexia.

The chapter on idiotcy and fatuity need not detain us.

### CHAP. VI.—TREATMENT.

This is usually considered the most important part of any medical subject though it will not always be found the most satisfactory—and for the reason stated at the very beginning of the chapter. "It is impossible to lay down any particular plan to be adopted in all cases." Yet it is this particular plan, which nine-tenths of readers are eagerly in pursuit of, while perusing a medical work on any particular malady! Our author acknowledges that even in cases where the greatest similarity exists in the disease, yet differences in constitution will demand modifications of treatment. In this chapter, however, he has endeavoured to make a classification of those cases in which the same system, modified by individual circumstances, may be adopted.

The brain or nervous system being considered, in all cases, as the seat of the disease termed insanity, the most obvious divisions will be according to the nature of the existing lesions. The subject is then divided into two classes—disordered function, and diseased structure; or incipient and chronic insanity. It need hardly be stated that a cure can only be expected in the first class. Once organic disease of the brain having occurred, palliation of the mental affection is the most that can be expected. In the great majority of post mortem examinations, the traces of vascular excitement are found in the brain—and therefore we may safely infer that, with few exceptions, such as great losses of blood, starvation, &c., an increased circulation is the first pathological condition of insanity, in all cases. The cause or causes of this state, our author thinks, must depend on irritation or over-exertion of the brain itself. In nine cases out of ten, pain is felt in the head, at the beginning. Bleeding and antiphlogistic measures may, for a time, arrest the progress of the malady, but, unless the causes, whether idiopathic or sympathetic, are removed, the malady will not be cured. Nay, copious and repeated depletions often do harm instead of good—and they are too often employed by young practitioners, under the idea that they have common phrenitis to deal with. When, however, insanity is caused by blows on the head, coup-de-soleil, or other similar agents, active depletion is obviously indicated, with all the other antiphlogistics well known in practice. It has been long observed that, when the brain is in a state of inordinate activity, the nervous energy will be found defective in some other parts or functions, they requiring immense doses of medicines to restore the equilibrium.

The MORAL TREATMENT of insanity is next taken up by our author. The first object is to remove the moral cause. But, alas! this is not often in our power. It does, however, sometimes come within our reach—as the presence of some object or objects that too much excite the brain and mind. Servants, on first coming to a lunatic asylum, are occasionally attacked with insanity themselves, and must be removed.

"When the exciting cause cannot be removed, the patient should be placed in circumstances calculated as much as possible to produce a complete interruption to the train of thought; every object at all likely by association to recall to the mind the painful circumstances, should be avoided; the patient ought to be surrounded with other objects. The usual routine of his habits ought to be broken in upon, and the attention attracted by a change in the little domestic arrangements; and, however painful, he should be at once withdrawn from the society of his friends. If the diseased action be but small, and the attack just in its commencement, I know of no means of accomplishing this more effectually than by sending the patient on an excursion into a fine country, mountainous if possible: the air, the scenery, and the exercise, all have a salutary influence; and the separation is by this means effected without causing any pain either to him or to his friends; but he ought, if possible, to be accompanied during the journey, by an experienced medical attendant; and the physical remedies for the relief of the brain ought to be most carefully attended to." 191.

Much disappointment has arisen from the want of this precaution. With all our partiality for travelling-exercise, even through the most beautiful and romantic countries on earth, we are compelled to admit that, in our experience, it has had but small and temporary influence on the mind verging towards insanity. It is more powerfully beneficial in the common corporeal ailments—especially in those connected with disorders of the chylopoietic viscera. Where insanity has actually taken place it is needless to observe that separation from friends, and location under medical superintendence, are essential to recovery.

"It is painful for friends to intrust their dearest relatives to strangers, and to run the risk on their recovery of being thought to have acted towards them harshly and precipitately; but unless they are willing to have the best interests of the sufferers sacrificed to a selfish caution and a foolish delicacy, they will not hesitate, however trying, to incur the responsibility of placing them, on the very commencement of the disease, where they will have an opportunity of receiving the best medical and moral treatment; and where they will at least be prevented from inflicting upon themselves, or those about them, any bodily injury." 192.

Many valuable lives are annually lost by this reluctance—natural but unwise—to sever the insane from their friends. But we are unable to dwell longer on this important chapter on the moral management of the insane. It ought to be studied carefully, especially by all those who devote their time and attention to the subject of insanity. We shall conclude with the following extract favourable to the science of phrenology.

"In the moral treatment of cases of insanity, it is of great importance to ascertain the ruling passion of the patient: an appeal to this will frequently divert the attention, and obviate the necessity of having recourse to violent measures. A female, of great firmness, had for several days refused to take her food, and as no persuasion seemed to have any influence upon her, preparations were made to inject it by the stomach-pump. At this juncture my wife discovered that the woman had naturally a great love of acquiring. She sat down by the patient's bedside, and without saying anything on the subject of food, conversed with her on her former habits; and having learnt that she had kept cows and poultry, she induced her to give an account of the profits she made by them. This attracted the attention of the woman: she forgot her determination to resist; and whilst talking of the gain of selling the butter, she permitted herself to be fed with a basin of bread and milk, apparently unconscious that she was submitting to the wishes of her attendants. In this instance phrenology was of practical use. The existence of the strong feeling of love of gain was ascertained solely by the observation of the head at the time. Another instance of the power of checking the violent operation of one set of feelings by calling another into action, also occurred to my wife. A patient, who was pruning some trees in the garden, quarrelled with another lunatic, during the accidental absence of the gardener: he became so irritated that he threatened to kill the other. A third patient ran into the house to give the alarm. He met my wife on the way, and she returned with him to the combatants, and desiring to speak with the man who had the knife, told him she was surprised to find a man, of his talents and understanding, so far forgetting himself as to dispute with the other, who, as he knew, had been insane for several years. This gratified his self-esteem. He said, You are right, ma'm; I shall take no farther notice of him;—and he at once became quiet." 221.

In this unpretending volume will be found a vast deal of highly important and useful practical information.

